# Activities of Antioxidant and Proteolytic Systems and Biomarkers in the Fat Body and Hemolymph of Young *Apis mellifera* Females

**DOI:** 10.3390/ani12091121

**Published:** 2022-04-27

**Authors:** Aneta Strachecka, Karolina Kuszewska, Krzysztof Olszewski, Patrycja Skowronek, Maciej Grzybek, Marcin Grabowski, Jerzy Paleolog, Michał Woyciechowski

**Affiliations:** 1 Department of Invertebrate Ecophysiology and Experimental Biology, University of Life Sciences in Lublin, Doświadczalna 50a, 20-280 Lublin, Poland; patrycja.skowronek@up.lublin.pl (P.S.); maciej.grzybek@gumed.edu.pl (M.G.); grabowski.entomologist@gmail.com (M.G.); jerzy.paleolog@up.lublin.pl (J.P.); 2 Institute of Environmental Sciences, Jagiellonian University, 30-387 Krakow, Poland; k.kuszewska@uj.edu.pl (K.K.); michal.woyciechowski@uj.edu.pl (M.W.); 3 Faculty of Animal Sciences and Bioeconomy, Institute of Biological Basis of Animal Production, University of Life Sciences in Lublin, Akademicka 13, 20-950 Lublin, Poland; krzysztof.olszewski@up.lublin.pl; 4 Department of Tropical Parasitology, Institute of Maritime and Tropical Medicine, Medical University of Gdansk, Powstania Styczniowego 9B, 81-519 Gdynia, Poland

**Keywords:** antioxidant, biomarkers, fat body, honeybee, hemolymph, inhibitor, protease, rebels, workers, queen

## Abstract

**Simple Summary:**

The proteolytic system consists of compounds that, similar to “scissors”, cut proteins found in bee cells (e.g., to activate these proteins) or released by pathogens. During these reactions, reactive oxygen species are created and then removed by antioxidants. The actions of the proteolytic and antioxidant systems are enhanced by biomarkers. These compounds are produced mainly in the fat body and then released into the hemolymph. We determined the activities of these compounds in various localizations/segments of the fat body and in the hemolymph in females with increased reproductive potential, i.e., queens and rebels, and in normal (sterile non-rebel) workers. Rebels are workers who resemble the queen in terms of anatomical, behavioural, and physiological features. It was revealed that the activities of these compounds in the rebels were between those of queens and normal workers. Normal workers had higher activities of the proteolytic and antioxidant systems in the fat body and hemolymph than the other females. These results are important for understanding the functioning of the fat body, the stress ecology, and the formation of the different castes of *Apis mellifera* females.

**Abstract:**

The proteolytic and antioxidant systems are important components of humoral immunity, and these biomarkers indicate the immune status. These compounds are synthesized in the bees’ fat body and released into the hemolymph. Their functions maintain the organism’s homeostasis and protect it against adverse environmental factors (including pathogens). We determined the activities of acidic, neutral, and alkaline proteases and their inhibitors, as well as superoxide dismutase (SOD), catalase (CAT), aspartate aminotransferase (AST), alanine aminotransferase (ALT), alkaline phosphatase (ALP), and the level of total antioxidant potential (TAC). These compounds were investigated in the fat body and hemolymph in the females with increased reproductive potential, i.e., queens and rebels, and in normal (non-reproductive sterile non-rebel) workers. The phenoloxidase (PO) activities were determined in the hemolymph. The normal workers had higher activities of proteases and their inhibitors, SOD and CAT, in the fat body and hemolymph, compared to the queens and rebels. The protease inhibitors were not usually active in the queens. As we predicted, the rebels revealed values between those of the queens and normal workers. The highest activities of proteases and antioxidants were identified in the fat body from the third tergite in comparison with the sternite and the fifth tergite. These results are important for oxidative stress ecology and give a better understanding of the functioning of the fat body and the division of labor in social insects.

## 1. Introduction

Honey bees are an essential component of the ecosystem and modern agriculture [1]. Pollination and plant–pollinator interactions are important for reproductive success and fruit production [2]. This ecosystem service supports the maintenance of biodiversity [1,2]. The worldwide economic value of pollination is estimated at €153 billion, to which honey bees are the principal contributors [1,3].

Unfortunately, as a result of the action of many factors, such as environmental pollution, monocultures, pathogens, or pesticides, bee immunity decreases, and the mortality of these beneficial insects rises [4]. Upon exposure to these foreign agents, the insect’s immune system, composed of highly developed cellular and humoral components, is activated [5]. These components work together to remove or neutralize the harmful factor so that the body returns to balance (homeostasis) [6]. Cellular responses are mediated by hemocytes in hemolymph, which are responsible for nodulation, encapsulation, and phagocytosis [5,6]. Moreover, together with the fat body cells, the hemocytes are involved in the mechanisms of the humoral response in which some of the first enzymes to be activated are those belonging to the proteolytic and antioxidant systems [6]. These systems complement each other and work in two directions: they are directed against harmful factors (e.g., pathogens) and/or enable the maintenance of homeostasis in apian cells and organisms by triggering many intracellular reactions and cascades (e.g., activating zymogens, hormones, etc.) [7,8].

Proteolytic enzymes act similarly to scissors and “cut” the pathogen’s proteins into smaller units [7]. These reactions generate reactive oxygen species, which are removed by antioxidants [8]. Moreover, intercellular proteolytic enzymes can recognize and preferentially degrade oxidatively damaged proteins to amino acids [7,8]. These systems are supported by biomarkers such as aspartate aminotransferase (AST), alanine aminotransferase (ALT), and alkaline phosphatase (ALP) [9]. Their decreased activities indicate the intensification of inflammatory and/or defence processes [9,10].

The synthesis of the compounds of these systems takes place in the bees’ fat bodies. Moreover, the fat body is responsible for the synthesis of other proteins, including those related to immunity, energy compounds, hormones, and pheromones [11,12]. Since the fat body has a segmental character and each segment works separately, and they all contribute to a common metabolism [13], various compounds are produced in different fat body locations and then released into the hemolymph [14]. We showed in our earlier publication [13] that the segmental character of the fat body corresponded with the caste reproductive potential and physiological characteristics shaped in the evolutionary process. An example would be the females with an increased reproductive potential characterized by the presence of oenocytes in the third tergite and high concentrations of compounds responsible for energy reserves, such as glucose, glycogen, and triglycerides [13]. Such females with an increased reproductive potential are queens and rebels [15]. The rebels are workers who have more ovarioles in the ovaries, less developed hypopharyngeal glands, and larger mandibular glands in comparison to normal (sterile non-rebel) workers [15]. They also develop immediately after swarming when the old queen has flown away with a swarm, and the new one is still in the pupa stage [15]. As rebels share many of the characteristics of queens and normal workers, it is hoped that understanding their physiology, morphology, and behavior will help understand the ways that led to the origin of different castes in females of eusocial Hymenoptera.

Since the first line of defence, composed of the proteolytic system, the antioxidant system, and biochemical markers, is a key antipathogenic barrier, we asked the question: will females with increased reproductive potential have higher activities of compounds belonging to these systems? It can be assumed that since such females have higher levels of the juvenile hormone, vitellogenin, and the phagocytic index [6], the activities of these compounds will also be higher. Therefore, the aim of our paper was to determine the activities of the proteolytic and antioxidant system as well as biochemical markers in the fat body and hemolymph in young queens, rebels, and normal (sterile non-rebel) workers.

## 2. Materials and Methods

This study was performed at the apiary of the University of Life Sciences in Lublin, Poland (51.224039° N-22.634649° E). Four colonies of *Apis mellifera* honey bees were used; three of them–the source colonies–were used to obtain larvae of known ages to rear normal workers and rebels; and one (colony 4) for rearing queens.

### 2.1. Experimental Design

A queen was taken from each of the three unrelated source colonies, each of which populated two-box hives (Dadant Blatt from Łysoń Beekeeping Company, Klecza Górna; 20 frames; 435 × 150 mm) and caged within a queen-excluder comb-cage containing two empty combs (C1 and C2) for egg laying for 24 h. On the third day after the end of egg-laying, 50 one-day-old (12–24-h-old) larvae from C1 and C2 were grafted into queen cell cups and suspended vertically in colony no. 4, according to Büchler et al.’s [16] method. After the larvae were grafted, C1 and C2 were restored to their source colonies with the remaining larvae. Next, each of the source colonies was divided into two equal parts, each in a separate box, according to Woyciechowski and Kuszewska’s [15] procedure. The first part (top box), containing the queen, workers, brood, and C1, was used for rearing normal (sterile; non-rebel) workers, whereas the other part (bottom box), without a queen but with workers, brood, and C2, served for rearing rebels. After sealing the larval cells in C1 and C2, the two boxes were put together again, respectively, so as to restore each of the three source colonies. After 15 days from the moment the eggs were laid, the sealed queen cells were placed in an incubator (temperature 34.5 °C, relative humidity 60%). After 18 days, the brood combs C1 and C2 were also placed in this incubator. Twenty normal workers and rebels from each of these combs and 60 queens were captured for hemolymph, fat body, and ovarian tubule sampling.

### 2.2. Hemolymph and Fat Body Collection

A glass capillary (20 µL; ‘end to end’ type; without anticoagulant; Medlab Products, Raszyn, Poland) was individually inserted between the third and fourth tergite of living normal workers or rebels or queens to obtain fresh hemolymph, according to Łoś and Strachecka’s [9] method. Hemolymph volumes were separately measured in each capillary. Hemolymph from one bee was collected into one sterile Eppendorf tube containing 25 µL of ice-cooled 0.6% NaCl. The hemolymph solution was immediately refrigerated at −80 °C for further biochemical analyses.

Immediately after collecting the hemolymph, the fat bodies from sternites (between the second and the fifth), the third and the fifth tergites (see Strachecka et al. [6,13]) in each of the 60 queens, 60 rebels and 60 normal workers were dissected under a Stereo Zoom Microscope. Each of the fat bodies was dissected and collected in sterile Eppendorf tubes containing 25 μL of ice-cooled 0.6% NaCl. Next, the tissues were homogenised at 4 °C and centrifuged for 1 min at 3000× *g*. The supernatants were immediately refrigerated at −40 °C for further biochemical analyses.

### 2.3. Biochemical Analyses

The activities of proteolytic and antioxidant systems and also biomarkers were determined in the hemolymph solutions and fat body supernatants.

#### 2.3.1. Determination of Proteolytic System Activities

- Proteolytic activity test in relation to substrates (gelatine, haemoglobin, ovalbumin, albumin, cytochrome C, casein): 1 µL of each supernatant was incubated with 2 µL of each of the six substrates (1%, *w*/*v*) in an appropriate buffer for 120 min at 37 °C. The reactions were ended by adding 8 µL of cold 5% trichloroacetic acid (TCA). The supernatant was analysed spectrophotometrically (Synergy HTX (S1LFA); Warsaw, Poland) to measure the absorbance at 280 nm. The results obtained in this way allowed us to choose haemoglobin as the optimal substrate for further analyses. For more methodological details, see the Anson method [17] modified by Strachecka et al. [18,19].
- The activities of acidic, neutral, and alkaline proteases were assayed in three buffers, respectively: 100 mM glycine–HCl at pH 2.4, 100 mM Tris–HCl at pH 7.0, and 100 mM glycine–NaOH at pH 11.2. Next, 1 µL of each supernatant was incubated with 2 µL of 1% (*w*/*v*) haemoglobin in an appropriate buffer for 90 min at 37 °C. The reactions were ended by adding 8 µL of cold 5% trichloroacetic acid (TCA); the undigested proteins were precipitated and centrifuged for 1 min at 17,709× *g* rcf. The supernatant was spectrophotometrically analysed to measure the absorbance at 280 nm. One unit of enzyme activity was defined as the number of enzymes producing a 0.001 increase in absorbance per minute, according to Anson [17]. For more methodological details, see the Anson method [17] modified by Strachecka et al. [18,19].
- Determination of the activities of natural inhibitors of acidic, neutral, and alkaline proteases, based on the Lee and Lin method [20]. Pepsin was used as a marker for acidic, whereas trypsin was used for neutral and alkaline proteases. Then, 1 µL of pepsin or trypsin (1 mg/mL) was preincubated with 1 µL of a given supernatant for 30 min at 37 °C. After this time, 5 µL of 1% haemoglobin in an appropriate buffer were added, and the incubation was continued for 60 min. The reactions were terminated by adding 12 µL of trichloroacetic acid (TCA), centrifuged for 1 min at 17,709× *g* rcf and the supernatants were spectrophotometrically analysed to measure the absorbance at 280 nm. Inhibitor activities were calculated according to Lee and Lin [20].
- Proteolytic activities after the addition of pepstatin A, PMSF, iodoacetamide, and o-phenanthroline (the diagnostic inhibitors): 1 µL of diagnostic inhibitors (2 mM) was preincubated with 1 µL of a given supernatant for 30 min at 37 °C. After this time, 5 µL of 1% haemoglobin in an appropriate buffer were added, and the incubation continued for 90 min. The reactions were ended by adding 12 µL of trichloroacetic acid (TCA), and the supernatants were measured as described above. Proteolytic activities after the addition of the diagnostic inhibitors were calculated according to the Lee and Lin method [20].

#### 2.3.2. Determination of Antioxidant System Activities

The antioxidant activities were measured with the kites:

- Superoxide dismutase (SOD) determined using a commercial Sigma-Aldrich (19,160) SOD Determination Kit (Poznań, Poland);
- Catalase (CAT) determined using a Catalase Assay Kit (219265-1KIT) from Sigma-Aldrich (Poznań, Poland);
- Total antioxidant capacity (TAC) determined using a Total Antioxidant Capacity Assay Kit (MAK187-1KT) from Sigma-Aldrich (Poznań, Poland).
- All antioxidant enzyme activities were calculated per 1 mg of protein.
- The phenoloxidase (PO) activities were determined according to the method used by Ptaszyńska et al. [21]. Two microliters of the hemolymph solution were added to 18 μL of TBS (Cayman Chemical, Ann Arbor, MI, USA), containing 5 mM CaCl_2_ in the wells of a 96-well plate. After 20 min of incubation at room temperature, 180 μL of 2 mM L-dihydroxyphenylalanine (L-DOPA) in 50 mM sodium phosphate, pH 6.5, was added. PO activity was determined spectrophotometrically on the basis of the amount of melanin formed (absorbance at 490 nm) over 60 min, at 2-min intervals, using the Synergy HTX (BioTek, Janki, Poland) microplate reader. The PO activities were determined in triplicate for each hemolymph solution.

#### 2.3.3. Determination of Biomarker Activities

The activities of aspartate aminotransferase (AST), alanine aminotransferase (ALT) and alkaline phosphatase (ALP) were measured with the kinetic method using monotests from Cormay (Lublin, Poland) according to the manufacturer’s procedure.

### 2.4. Examination of Anatomical Characteristics

In order to confirm whether the emerging bees were rebels or normal workers, as well as queen status, Woyciechowski and Kuszewska’s [15] method was used to determine the number of ovarioles (ovarian tubules). The total number of ovarioles in both ovaries of each individual was counted. All ovarioles were stained with the Giemsa reagent (Sigma-Aldrich, Poznań, Poland) for approximately 10 s before being measured.

### 2.5. Statistical Analysis

The results were analyzed statistically using Statistica formulas (TIBCO Software, Palo Alto, CA, USA), version 13.3 (2017) for Windows–StatSoft Inc., Tulsa, OK, USA. The distribution of the data was analyzed with the Shapiro–Wilk test. To compare the number of ovarioles and biochemical parameters between the rebels, normal workers, and queens, a mixed-model two-way and three-way ANOVA was used. The experimental group was a fixed effect, and the colony was a random effect. If a difference among the groups was statistically significant, the ANOVA procedure was followed with multiple comparison testing using the post hoc Tukey HSD test with *p* = 0.05 as the level of significance.

## 3. Results

The values identified for the activities of proteases and their inhibitors in the rebels were always in between those in queens and workers regardless of the tissue type localisation (Table 1). Protease activities in all the females were the highest in the third tergites as compared to the other tissue type/localisation. The acidic and neutral protease inhibitors were not active in various localisations of the fat body and in the hemolymph of the queens, while alkaline protease inhibitors were not active only in the fat body; but on the other hand, in the hemolymph, their values were very high.

Aspartic, serine, thiolic and metallic proteases were observed in all the tissue types/localisations in the females after adding pepstatin A, PMSF, iodoacetamide, and o-phenanthroline (the diagnostic inhibitors).

The activities of SOD and CAT were the highest among the normal workers in comparison to the queens and rebels (Table 2). SOD activities in the queens were at the same levels in all tissue types/localisations. SOD and CAT had the highest activities in the hemolymph as compared to the other tissue types/localisations (the exception was SOD in the queens).

The TAC levels in the queens were the highest in the third tergite of the fat body as compared to the other tissue types/localisations (Table 2). In the case of the rebels and normal workers, the highest values were observed in the hemolymph.

**Figure 1:**
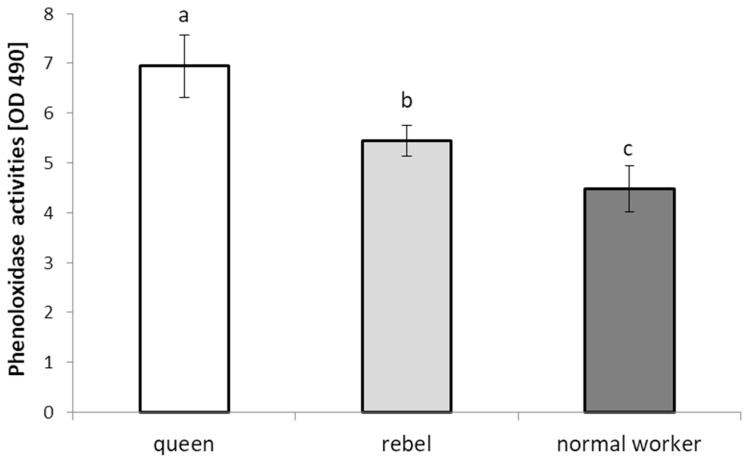
Phenoloxidase activities in the hemolymph of the *A. mellifera* queens, rebels, and normal (sterile non-rebel) workers. Lowercase letters indicate significant differences between phenotypes (queen/rebel/normal worker) with *p* < 0.05.

The PO activities were the highest in the queens in comparison to the rebels and normal workers (Figure 1).

The queens had the highest activities of all biomarkers (Table 3) as compared to the rebels and normal workers. The highest AST activities were found in the hemolymph of all the females. In the case of ALP, these values were the highest in the sternites of all the phenotypes. ALT activities were always the lowest in the third tergites as compared to the other tissue types/localisations.

We confirmed that the females were queens, rebels, or normal workers (Table 4). The highest number of ovarioles was observed in the queens and the lowest–in the normal workers.

## 4. Discussion

The proteolytic and antioxidant systems form the biochemical immune system of bees, which is a component of humoral immunity. These systems are supported by biomarkers, which are signals of the immune status [5]. The females with increased reproductive potential had lower activities of proteases and their inhibitors compared to the normal workers (Table 1). Moments after emerging, workers began to perform various activities for the colony. Their mechanisms of resistance, especially the biochemical one through active enzymes, must be functional from the moment of exiting the comb cell. After emergence, the queen is cared for by workers who lick, feed, and protect her. There are over 80 proteins and phosphoproteins in the salivary glands and saliva, which are involved in many regulatory, metabolic, and physiological processes, including those related to immunity [22]. It is most likely because of this that the biochemical resistance of the queen is activated only before the mating flight [23]. It can be assumed that this is one of the mechanisms of adaptation to unfavourable environmental factors. This is also confirmed in the research of Strachecka et al. [19], who showed that mature queens have fully activated enzymes of the proteolytic system. Matsuoka et al. [24] showed that queen bee larvae have proteases with carboxypeptidase A-like and chymotrypsin-like activities. These proteases are used in the hydrolytic activation of enzymes in the larvae and royal jelly. At the pupal stage, these enzymes are deactivated, and perhaps this is why the one-day-old queens had lower protease (Table 1) activities than the workers. The effects of an inactive proteolytic system in queens are evident in the activities of other metabolically immune-related compounds. An example is phenoloxidase, which is activated by proteases, mainly serine proteases. The more active proteases are, the more the activity of the phenoloxidase increases. This tendency is also visible in our research (compare with Figure 1) and that of Brandt et al. [25] and Schmid et al. [26]. Medina et al. [27] observed that phenoloxidase activities were higher in Africanized honey bee workers as compared to reproductive queens of the species. Moreover, pro-phenoloxidase is regulated (activation or inhibition) by protease inhibitors and by the presence of microbial-origin compounds, such as 1,3-β-glucans, lipopolysaccharides, and peptidoglycans [28]. Active phenoloxidase catalyzes reactions in the melanization and sclerotization pathways that seal and protect the bees against the ingress of pathogens and other harmful factors [29]. Activating all these processes in young workers is essential for their health and homeostasis [28]. In most cases, the activities of the proteolytic system, phenoloxidase, and other antipathogenic proteins and enzymes in *A. mellifera* workers increase with their age [28,30,31,32]. In the case of queen bees, the mating flight is a strategic moment during which the female is in contact with the extra-hive environment and many stressors [33].

It should be noted that the increase in the female reproductive status corresponds with a decline in the activities of protease inhibitors in the fat body and in the hemolymph, which is the case with queens. Acidic protease inhibitors are directed primarily against pathogenic fungi, basic against bacteria and viruses, and neutral against other agents [6,34]. It can be assumed that the young queen does not need acidic and neutral inhibitors in the fat body and hemolymph, as well as alkaline protease inhibitors in the fat body, and has other mechanisms that protect her from negative factors [34,35]. In turn, the high activities of alkaline protease inhibitors in the hemolymph of these queens are related not only to the regulation of intracellular specific proteases but also to the regulation of phenoloxidase activation, cellular response (e.g., phagocytosis), vitellogenin levels, etc. [6,34,35,36].

We observed particularly high protease activities in the third tergite of the fat body of female bees. Our previous research [13] showed that this segment is crucial, especially for females with increased reproductive potential. First of all, the fat body in this localization does not contain oenocytes but only trophocytes, which accumulate very large amounts of energy compounds [13]. Secondly, this is the most likely synthetisation site of vitellogenin, which is essential in maintaining the reproductive status and in the processes related to antioxidation and immunity of queens and rebels [6,13,24].

During various reactions in metabolic processes, reactive oxygen species (ROS) are formed and then inactivated by the antioxidant system [8,32]. Paleolog et al. [8] showed that there is an inverse relationship in the activities of antioxidants in queens and workers in certain periods of their lives (young females versus old females). Comparing the results of antioxidant activities in the hemolymph from the publications of Paleolog et al. [8] and ours, we conclude that they are consistent. SOD activities were always higher in normal workers (Table 2). In contrast, the CAT activities in the hemolymph were opposite, i.e., higher in young queens. Activities of SOD and CAT in the fat body (in three localisations) were always higher in normal workers. The values of these enzymes in rebels were intermediate, at levels between those of queens and normal workers. To the best of our knowledge, our results are the first to present the antioxidant system in the segments of the subcuticular fat body of *A. mellifera* females, i.e., its differentiation depending on the abdominal segment in which it is present. This finding is important for oxidative stress ecology [37] and a better understanding of the division of labour in social insect colonies [38,39]. Hsu and Hsieh [40] showed that CAT activities increased with age, whereas SOD activities decreased with age in the fat body of workers. However, these researchers do not specify which regions of the body the fat body emerges. It must be stressed that there are different parts of the fat body: visceral and subcuticular, which differ in structure and function [41]. Assuming they determined antioxidant activities in the subcuticular fat body, our research shows that these values depend on the localisation/segment of the fat body and the bee caste/subcaste/phenotype. The antioxidant system consists of many enzymatic and non-enzymatic elements that are related to the action of other compounds, including those in the proteolytic system, juvenile hormones, vitellogenin, energetic compounds, and lipids [42], which have different expression levels in normal workers and queens and rebels [6,13,43]. The ratio of saturated to unsaturated fats in cell membranes, which promotes the resistance of bees to oxidative stress [44,45], should be taken into consideration as well. Vitellogenin is also a strong antioxidant [46] with lower expression in workers as compared to queens and rebels [6,47]. Different antioxidant activities in the phenotypes of bee females may be the result of differences in the expression of antioxidant genes during the individual development of these castes [48].

Biomarkers (AST, ALT, and ALP) are the basic compounds informing the physiological state of bees [9]. Unlike in mammals, the high activities of these compounds in female bees signal their good condition, vitality, and immunity [9,32]. The activities of AST, ALT, and ALP were always higher in the females with increased reproductive potential as compared to the normal workers (Table 3). It can be assumed that high biomarker activities in queens and rebels complement and stabilize the functioning of their proteolytic systems. The compound activities decreased in reaction to harmful factors, i.e., antibiotics, acaricides, electric field, *Varroa destructor*, etc. [9,10]. Low activities of the enzymes in bees may result in changes in the Krebs cycle, ATP synthesis, oxidative phosphorylation, β-oxidation, and other metabolic cycles and may also indicate the presence of low protein concentrations in the diet [49,50]. High values of these biomarkers were observed in the sternite and hemolymph. It can be presumed that it is the sternite fat body that performs the function of the “bee liver”.

## 5. Conclusions

Although they are synthesised in the fat body [13], proteolytic and antioxidant systems did not always show activity in their individual localisations/segments. Examples were the inhibitors of acidic, neutral, and alkaline proteases in the queens. The normal (sterile non-rebel) workers were characterised by high activities of protease inhibitors, SOD and CAT, and low activities of biomarkers. This proves their adaptation to the functions performed in the bee colony. Our research shows that most of the physiological parameters in rebels are intermediate between those of queens and normal workers. Moreover, we presented for the first time the values of these biochemical parameters in specific segments/localisations in the fat body, which further confirms the segmental nature of this tissue. Our findings can help better understand the ways that led to the origin of different castes/sub-castes/phenotypes in the females of eusocial Hymenoptera and the functioning of the fat body in each of the female types. Moreover, the results are important for oxidative stress ecology and a better understanding of the division of labour in social insect colonies.

## Figures and Tables

**Table 1:** Activities of the proteolytic system in the fat body and hemolymph of the *A. mellifera* queens, rebels, and normal (sterile non-rebel) workers.

		Queen		Rebel		Normal Workers		Queen		Rebel		Normal Workers		Queen		Rebel		Normal Workers	
		Mean	SD	Mean	SD	Mean	SD	Mean	SD	Mean	SD	Mean	SD	Mean	SD	Mean	SD	Mean	SD
		Acidic Proteases (U/mg)						Neutral Proteases (U/mg)						Alkaline Proteases (U/mg)					
**tissues**	**sternit**	0.61	0.05	1.00	0.06	1.25	0.08	0.35	0.04	0.44	0.03	1.26	0.08	2.86	0.33	2.96	0.14	3.37	0.19
	**tergit 3**	3.25	0.33	3.55	0.16	4.34	0.18	3.07	0.25	3.42	0.20	4.19	0.19	3.26	0.21	3.51	0.21	4.42	0.22
	**tergit 5**	1.43	0.10	1.34	0.04	1.17	0.05	2.44	0.16	2.30	0.10	1.99	0.10	2.51	0.14	2.40	0.06	2.19	0.08
	**hemolymph**	0.75	0.07	0.84	0.03	1.06	0.04	0.76	0.08	0.92	0.04	2.30	0.14	0.96	0.06	0.82	0.03	0.06	0.00
**Three-Way Anova**	**colony**	F_(2,684)_ = 1.50; *p* = 0.492						F_(2,684)_ = 7.75; *p* = 0.80						F_(2,684)_ = 0.41; *p* = 0.695					
	**phenotype**	F_(2,4)_ = 123.98; *p* = 0.0003						F_(2,4)_ = 555.98; *p* = 0.000						F_(2,4)_ = 8.18; *p* = 0.038					
	**tissue**	F_(3,6)_ = 4019.96; *p* = 0.000						F_(3,6)_ = 5447.52; *p* = 0.000						F_(3,6)_ = 1285.66; *p* = 0.000					
	colony * phenotype	F_(4,12)_ = 0.89; *p* = 0.501						F_(4,12)_ = 0.68; *p* = 0.618						F_(4,12)_ = 0.70; *p* = 0.607					
	colony * tissue	F_(6,12)_ = 0.74; *p* = 0.631						F_(6,12)_ = 0.459; *p* = 0.826						F_(6,12)_ = 1.69; *p* = 0.206					
	phenotype * tissue	F_(6,12)_ = 47.82; *p* = 0.000						F_(6,12)_ = 113.15; *p* = 0.000						F_(6,12)_ = 92.98; *p* = 0.000					
	colony * phenotype * tissue	F_(12,684)_ = 8.10; *p* = 0.000						F_(12,684)_ = 7.031; *p* = 0.000						F_(12,684)_ = 6.34; *p* = 0.000					
		**acidic protease inhibitors (U/mg)**						**neutral protease inhibitors (U/mg)**						**alkaline protease inhibitors (U/mg)**					
**tissues**	**sternit**	0	0	0.03	0.00	0.25	0.03	0	0	0.02	0.00	0.13	0.02	0	0	0.02	0.02	0.24	0.25
	**tergit 3**	0	0	0.15	0.02	0.54	0.03	0	0	0.01	0.00	0.24	0.03	0	0	0.03	0.04	0.34	0.35
	**tergit 5**	0	0	0.14	0.02	0.33	0.02	0	0	0.01	0.00	0.14	0.02	0	0	0.03	0.03	0.33	0.34
	**hemolymph**	0	0	3.78	0.29	3.21	0.15	0	0	2.89	0.11	2.34	0.14	5.26	0.47	3.92	0.12	3.55	0.17
**Three-Way Anova**	**colony**	F_(2,456)_ = 1.03; *p* = 0.675						F_(2,456)_ = 3.30; *p* = 0.675						F_(2,456)_ = 0.639; *p* = 0.608					
	**phenotype**	F_(1,2)_ = 8.93; *p* = 0.096						F_(1,2)_ = 4.0; *p* = 0.184						F_(2,4)_ = 108.83; *p* = 0.001					
	**tissue**	F_(3,6)_ = 11,659.16; *p* = 0.000						F_(3,6)_ = 11,257.50; *p* = 0.000						F_(3,6)_ = 71,825.71; *p* = 0.000					
	colony * phenotype	F_(2,6)_ = 1.03; *p* = 0.412						F_(2,6)_ = 0.959; *p* = 0.435						F_(2,6)_ = 2.90; *p* = 0.118					
	colony * tissue	F_(6,12)_ = 0.56; *p* = 0.752						F_(6,12)_ = 0.105; *p* = 0.992						F_(6,12)_ = 0.256; *p* = 0.939					
	phenotype * tissue	F_(3,6)_ = 113.73; *p* = 0.000						F_(3,6)_ = 241.70; *p* = 0.000						F_(3,6)_ = 224.79; *p* = 0.000					
	colony * phenotype * tissue	F(_6,456)_ = 3.82; *p* = 0.001						F_(6,456)_ = 3.761; *p* = 0.001						F_(6,570)_ = 1.19; *p* = 0.304					

Tergit 3—the fat body from the third tergite; tergite 5—the fat body from the fifth tergite; *—interaction.

**Table 2:** Activities of the antioxidant system in the fat body and hemolymph of the *A. mellifera* queens, rebels, and normal (sterile non-rebel) workers.

		Queen		Rebel		Normal Workers		Queen		Rebel		Normal Workers		Queen		Rebel		Normal Workers	
		Mean	SD	Mean	SD	Mean	SD	Mean	SD	Mean	SD	Mean	SD	Mean	SD	Mean	SD	Mean	SD
		SOD (U/mg)						CAT (U/mg)						TAC (mM of Trolox)					
**tissues**	**sternit**	0.44	0.03	0.48	0.04	0.75	0.03	1.21	0.09	1.16	0.02	2.28	0.24	1.26	0.12	1.48	0.04	3.26	0.19
	**tergit 3**	0.44	0.03	0.49	0.03	0.55	0.03	0.84	0.05	1.03	0.10	1.16	0.04	11.78	0.98	10.22	0.40	9.49	0.39
	**tergit 5**	0.44	0.03	0.46	0.03	0.64	0.03	1.07	0.13	1.20	0.14	1.62	0.05	1.55	0.20	3.12	0.22	3.47	0.23
	**hemolymph**	0.44	0.03	0.60	0.05	1.15	0.05	7.41	0.45	7.11	0.18	5.38	0.23	3.61	0.24	48.07	0.65	45.54	0.51
**Three-Way Anova**	**colony**	F_(2,684)_ = 0.12; *p* = 0.886						F_(2,684)_ = 18.43; *p* = 0.695						F_(2,684)_ = 0.852; *p* = 0.740					
	**phenotype**	F_(2,4)_ = 4786.06; *p* = 0.000						F_(2,4)_ = 0.18; *p* = 0.840						F_(2,4)_ = 5486.0; *p* = 0.000					
	**tissue**	F_(3,6)_ = 1198.62; *p* = 0.000						F_(3,6)_ = 25,638.1; *p* = 0.000						F_(3,6)_ = 21,196.0; *p* = 0.000					
	colony * phenotype	F_(4,12)_ = 2.56; *p*=0.092						F_(4,12)_ = 0.765; *p* = 0.56						F_(4,12)_ = 0.7; *p* = 0.632					
	colony * tissue	F_(6,12)_ = 2.89; *p* = 0.055						F_(6,12)_ = 0.28; *p* = 0.935						F_(6,12)_ = 0.6; *p* = 0.701					
	phenotype * tissue	F_(6,12)_ = 2002.14; *p* = 0.000						F_(6,12)_ = 189.02; *p* = 0.000						F_(6,12)_ = 3445.0; *p* = 0.000					
	colony * phenotype * tissue	F_(12,684)_ = 0.48; *p* = 0.927						F_(12,684)_ = 5.99; *p* = 0.000						F_(12,684)_ = 23.5; *p* = 0.000					

Tergit 3—the fat body from the third tergite; tergite 5—the fat body from the fifth tergite; SOD—superoxide dismutase; CAT—catalase; TAC—total antioxidant capacity; *—interaction.

**Table 3:** Activities of biomarkers in the fat body and hemolymph of the *A. mellifera* queens, rebels, and normal (sterile non-rebel) workers.

		Queen		Rebel		Normal Workers		Queen		Rebel		Normal Workers		Queen		Rebel		Normal Workers	
		Mean	SD	Mean	SD	Mean	SD	Mean	SD	Mean	SD	Mean	SD	Mean	SD	Mean	SD	Mean	SD
		AST (U/dm^3^)						ALT (U/dm^3^)						ALP (U/dm^3^)					
**tissues**	**sternit**	25.4	0.36	22.3	0.46	21.7	0.39	30.3	0.53	25.3	0.63	24.4	0.47	7.5	0.24	6.29	0.43	5.44	0.26
	**tergit 3**	16.4	0.41	15.2	0.45	12.5	0.37	22.4	0.40	21.2	0.45	19.5	0.38	4.33	0.23	3.26	0.27	2.37	0.19
	**tergit 5**	12.2	1.32	12	0.38	10.6	0.28	25.5	0.26	24.7	0.35	20.7	0.42	3.81	0.17	2.68	0.20	1.85	0.05
	**hemolymph**	26.4	0.37	23.5	0.30	22.4	0.49	28.5	0.45	25.9	0.40	25.5	0.39	6.53	0.24	5.52	0.23	5.44	0.30
**Three-Way Anova**	**colony**	F_(2,684)_ = 1.4; *p* = 0.537						F_(2,684)_ = 0.4; *p* = 0.2						F_(2,684)_ = 0.9; *p* = 0.469					
	**phenotype**	F_(2,4)_ = 1787.9; *p* = 0.000						F_(2,4)_ = 7450; *p* = 0.000						F_(2,4)_ = 1261.26; *p* = 0.000					
	**tissue**	F_(3,6)_ = 64,287.6; *p* = 0.000						F_(3,6)_ = 12,184; *p* = 0.000						F_(3,6)_ = 7220.73; *p* = 0.000					
	colony * phenotype	F_(4,12)_ = 1.3; *p* = 0.325						F_(4,12)_ = 0.0; *p* = 0.773						F_(4,12)_ = 2.83; *p* = 0.073					
	colony * tissue	F_(6,12)_ = 0.4; *p* = 0.879						F_(6,12)_ = 0.0; *p* = 0.902						F_(6,12)_ =1.51; *p* = 0.255					
	phenotype * tissue	F_(6,12)_ = 146.3; *p* = 0.000						F_(6,12)_ = 254; *p* = 0.000						F_(6,12)_ = 67.31; *p* = 0.000					
	colony * phenotype * tissue	F_(12,684)_ = 1.0; *p* = 0.461						F_(12,684)_ = 2; *p* = 0.07						F_(12,684)_ = 0.87; *p* = 0.575					

Tergit 3—the fat body from the third tergite; tergite 5—the fat body from the fifth tergite; AST—aspartate aminotransferase; ALT—alanine aminotransferase; ALP—alkaline phosphatase; *—interaction.

**Table 4:** Numbers of ovarioles in the *A. mellifera* queens, rebels, and normal (sterile non-rebel) workers.

		Queen		Rebel		Normal Workers	
		Mean	SD	Mean	SD	Mean	SD
	**Ovariole number**	199.55	25.36	12.37	1.84	5.08	1.07
**Two-Way Anova**	**colony**	F_(2,4)_ = 1.002; *p* = 0.444					
	**phenotype**	F_(2,4)_ = 1442.48; *p* = 0.000					
	colony * phenotype	F_(4,171)_ = 2.458; *p* = 0.047					

*—interaction.

## Data Availability

The datasets generated during and/or analyzed during the current study are available from the corresponding author on reasonable request.
